# Bis[1,5-bis­(1*H*-indol-3-ylmethyl­ene)thio­carbazonato-κ^2^ *N*,*S*]nickel(II) dimethyl sulfoxide disolvate

**DOI:** 10.1107/S1600536808011975

**Published:** 2008-05-03

**Authors:** Mohd. Razali Rizal, Hapipah M. Ali, Seik Weng Ng

**Affiliations:** a Department of Chemistry, University of Malaya, 50603 Kuala Lumpur, Malaysia

## Abstract

The Ni atom in the crystal structure of the centrosymmetric title compound, [Ni(C_19_H_15_N_6_S)_2_]·2C_2_H_6_OS, is *N*,*S*-chelated by the deprotonated Schiff bases in a square-planar geometry. The –CH=N—N=C(S)—NH—N=CH– frament is planar. The two indolyl –NH (donor) sites inter­act with dimethyl sulfoxide mol­ecules to furnish a layer motif.

## Related literature

For the structure of the unsolvated nickel derivative of 1*H*-indole-3-carboxaldehyde thio­semicarbazone, see: Rizal *et al.* (2008 ▶). The ligand is known to be a sensitive complexing agent, see: Ghosh *et al.* (1999 ▶).
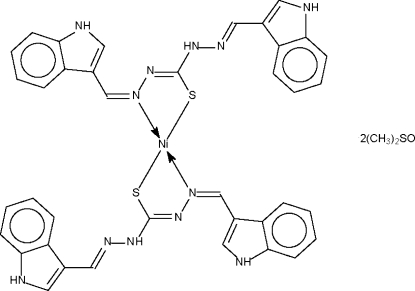


## Experimental

### 

#### Crystal data

[Ni(C_19_H_15_N_6_S)_2_]·2C_2_H_6_OS
*M* *_r_* = 933.83
Monoclinic, 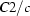

*a* = 19.0340 (5) Å
*b* = 9.1982 (3) Å
*c* = 25.1374 (7) Å
β = 95.672 (2)°
*V* = 4379.5 (2) Å^3^
*Z* = 4
Mo *K*α radiation
μ = 0.69 mm^−1^
*T* = 100 (2) K
0.30 × 0.03 × 0.03 mm

#### Data collection

Bruker SMART APEX diffractometer
Absorption correction: multi-scan (*SADABS*; Sheldrick, 1996 ▶) *T*_min_ = 0.821, *T*_max_ = 0.980
27119 measured reflections
5030 independent reflections
3201 reflections with *I* > 2σ(*I*)
*R*_int_ = 0.092

#### Refinement

*R*[*F* ^2^ > 2σ(*F* ^2^)] = 0.048
*wR*(*F* ^2^) = 0.139
*S* = 1.03
5030 reflections
277 parameters
H-atom parameters constrained
Δρ_max_ = 0.60 e Å^−3^
Δρ_min_ = −0.53 e Å^−3^

### 

Data collection: *APEX2* (Bruker, 2007 ▶); cell refinement: *SAINT* (Bruker, 2007 ▶); data reduction: *SAINT*; program(s) used to solve structure: *SHELXS97* (Sheldrick, 2008 ▶); program(s) used to refine structure: *SHELXL97* (Sheldrick, 2008 ▶); molecular graphics: *X-SEED* (Barbour, 2001 ▶); software used to prepare material for publication: *publCIF* (Westrip, 2008 ▶).

## Supplementary Material

Crystal structure: contains datablocks global, I. DOI: 10.1107/S1600536808011975/bt2702sup1.cif

Structure factors: contains datablocks I. DOI: 10.1107/S1600536808011975/bt2702Isup2.hkl

Additional supplementary materials:  crystallographic information; 3D view; checkCIF report

## Figures and Tables

**Table 1:** Selected bond lengths (Å)

Ni1—N5	1.906 (3)
Ni1—S1	2.1748 (8)

**Table 2:** Hydrogen-bond geometry (Å, °)

*D*—H⋯*A*	*D*—H	H⋯*A*	*D*⋯*A*	*D*—H⋯*A*
N1—H1n⋯O1	0.88	2.10	2.890 (4)	148
N6—H6n⋯O1^i^	0.88	2.03	2.855 (4)	156

Symmetry code: (i) 
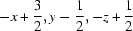
.

**Fig. 1.**
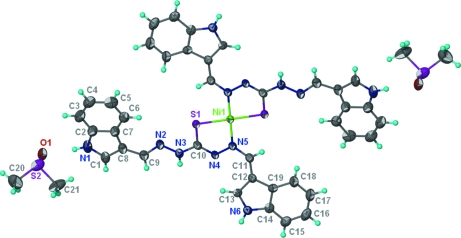
Thermal ellipsoid plot (Barbour, 2001) of Ni(C19H15N6S)2.2DMSO at the 70% probability level; hydrogen atoms are drawn as spheres of arbitrary radius. The molecule lies on a center-of-inversion. Unlabeled atoms are related to the labeled ones by this symmetry element.

## supplementary crystallographic information

### Comment

The preceding study reports the nickel derivative of 1*H*-indole-3-carboxaldehyde thiosemicarbazone (Rizal *et al.*, 2008). With bis(1*H*-indole-3-carboxaldehyde thiocarbazone) in place of the thiosemicarbazone, the resulting nickel derivative also has the *N*,*S*-chelated metal center in a square planar coordination geometry. The compound crystallizes from DMSO as a disolvate (Fig. 1). The oxygen atom of the solvent molecule is a hydrogen bond acceptor to the indolyl amino group of two mononuclear molecules; such a hydrogen bonding scheme gives rise to a layer motif.

### Experimental

The Schiff base was synthesized as according to a literature procedure (Ghosh *et al.*, 1999). The Schiff base (2 g, 5.5 mmol) and nickel acetate (0.7 g, 2.8 mmol) were heated in ethanol (50 ml) for 5 h. The brown product was recrystallized from DMSO to give red crystals..

### Refinement

Carbon-bound H-atoms were placed in calculated positions (C—H 0.98 Å) and were included in the refinement in the riding model approximation, with *U*(H) set to 1.2–1.5*U*(C). The nitrogen-bound H-atoms were similarly treated [N–H 0.88 Å].

### Crystal data

**Table:**

[Ni(C_19_H_15_N_6_S)_2_]·2C_2_H_6_OS	*F*_000_ = 1944
*M**_r_* = 933.83	*D*_x_ = 1.416 Mg m^−^^3^
Monoclinic, *C*2/*c*	Mo *K*α radiation λ = 0.71073 Å
Hall symbol: -C 2yc	Cell parameters from 2255 reflections
*a* = 19.0340 (5) Å	θ = 2.5–23.1º
*b* = 9.1982 (3) Å	µ = 0.69 mm^−^^1^
*c* = 25.1374 (7) Å	*T* = 100 (2) K
β = 95.672 (2)º	Needle, red
*V* = 4379.5 (2) Å^3^	0.30 × 0.03 × 0.03 mm
*Z* = 4	

### Data collection

**Table:**

Bruker SMART APEX diffractometer	5030 independent reflections
Radiation source: fine-focus sealed tube	3201 reflections with *I* > 2σ(*I*)
Monochromator: graphite	*R*_int_ = 0.092
*T* = 100(2) K	θ_max_ = 27.5º
φ and ω scans	θ_min_ = 1.6º
Absorption correction: multi-scan(SADABS; Sheldrick, 1996)	*h* = −24→24
*T*_min_ = 0.821, *T*_max_ = 0.980	*k* = −11→9
27119 measured reflections	*l* = −32→32

### Refinement

**Table:**

Refinement on *F*^2^	Secondary atom site location: difference Fourier map
Least-squares matrix: full	Hydrogen site location: inferred from neighbouring sites
*R*[*F*^2^ > 2σ(*F*^2^)] = 0.048	H-atom parameters constrained
*wR*(*F*^2^) = 0.139	*w* = 1/[σ^2^(*F*_o_^2^) + (0.0695*P*)^2^] where *P* = (*F*_o_^2^ + 2*F*_c_^2^)/3
*S* = 1.04	(Δ/σ)_max_ = 0.001
5030 reflections	Δρ_max_ = 0.60 e Å^−^^3^
277 parameters	Δρ_min_ = −0.53 e Å^−^^3^
Primary atom site location: structure-invariant direct methods	Extinction correction: none

### Special details

**Table:**

Geometry. All e.s.d.'s (except the e.s.d. in the dihedral angle between two l.s. planes) are estimated using the full covariance matrix. The cell e.s.d.'s are taken into account individually in the estimation of e.s.d.'s in distances, angles and torsion angles; correlations between e.s.d.'s in cell parameters are only used when they are defined by crystal symmetry. An approximate (isotropic) treatment of cell e.s.d.'s is used for estimating e.s.d.'s involving l.s. planes.

### Fractional atomic coordinates and isotropic or equivalent isotropic displacement parameters (Å^2^)

**Table:**

	*x*	*y*	*z*	*U*_iso_*/*U*_eq_	
Ni1	0.7500	0.7500	0.5000	0.02112 (17)	
S1	0.71406 (4)	0.69712 (10)	0.41745 (3)	0.0248 (2)	
S2	0.54373 (5)	0.32873 (12)	0.03925 (4)	0.0426 (3)	
N1	0.67553 (16)	0.4672 (4)	0.14187 (11)	0.0407 (8)	
H1N	0.6749	0.4384	0.1084	0.049*	
N2	0.73446 (14)	0.5251 (3)	0.32226 (10)	0.0275 (6)	
N3	0.77036 (14)	0.4729 (3)	0.36844 (10)	0.0278 (6)	
H3N	0.7967	0.3945	0.3674	0.033*	
N4	0.80017 (13)	0.4836 (3)	0.45745 (9)	0.0228 (6)	
N5	0.79004 (13)	0.5599 (3)	0.50425 (9)	0.0219 (6)	
N6	0.87026 (13)	0.1142 (3)	0.54254 (10)	0.0256 (6)	
H6N	0.8770	0.0305	0.5269	0.031*	
O1	0.61523 (12)	0.3930 (3)	0.03531 (9)	0.0361 (6)	
C1	0.71111 (19)	0.3994 (5)	0.18458 (13)	0.0366 (9)	
H1	0.7380	0.3128	0.1830	0.044*	
C2	0.64128 (19)	0.5856 (4)	0.15846 (13)	0.0347 (9)	
C3	0.59816 (19)	0.6863 (5)	0.13031 (15)	0.0410 (10)	
H3	0.5891	0.6801	0.0925	0.049*	
C4	0.5688 (2)	0.7948 (5)	0.15795 (16)	0.0437 (10)	
H4	0.5390	0.8642	0.1390	0.052*	
C5	0.5817 (2)	0.8061 (5)	0.21364 (16)	0.0445 (10)	
H5	0.5606	0.8827	0.2318	0.053*	
C6	0.62496 (18)	0.7069 (4)	0.24255 (14)	0.0344 (9)	
H6	0.6332	0.7142	0.2804	0.041*	
C7	0.65623 (18)	0.5962 (4)	0.21512 (12)	0.0314 (8)	
C8	0.70192 (18)	0.4760 (4)	0.23012 (12)	0.0321 (8)	
C9	0.73595 (18)	0.4385 (4)	0.28191 (12)	0.0307 (8)	
H9	0.7600	0.3482	0.2866	0.037*	
C10	0.76516 (16)	0.5425 (4)	0.41603 (12)	0.0237 (7)	
C11	0.80984 (15)	0.4880 (4)	0.54786 (12)	0.0239 (7)	
H11	0.8051	0.5388	0.5802	0.029*	
C12	0.83747 (16)	0.3449 (4)	0.55418 (12)	0.0237 (7)	
C13	0.84099 (16)	0.2324 (4)	0.51784 (12)	0.0235 (7)	
H13	0.8250	0.2380	0.4809	0.028*	
C14	0.88816 (16)	0.1434 (4)	0.59618 (13)	0.0274 (8)	
C15	0.92233 (17)	0.0566 (4)	0.63616 (13)	0.0346 (9)	
H15	0.9361	−0.0403	0.6293	0.042*	
C16	0.93510 (19)	0.1182 (5)	0.68610 (14)	0.0410 (10)	
H16	0.9589	0.0626	0.7142	0.049*	
C17	0.91418 (19)	0.2594 (5)	0.69662 (14)	0.0400 (9)	
H17	0.9234	0.2975	0.7317	0.048*	
C18	0.88050 (17)	0.3447 (4)	0.65717 (12)	0.0330 (9)	
H18	0.8661	0.4408	0.6647	0.040*	
C19	0.86766 (16)	0.2867 (4)	0.60527 (13)	0.0252 (7)	
C20	0.5318 (2)	0.1994 (5)	−0.01409 (19)	0.0615 (13)	
H20A	0.5247	0.2508	−0.0483	0.092*	
H20B	0.4904	0.1390	−0.0096	0.092*	
H20C	0.5737	0.1374	−0.0136	0.092*	
C21	0.5542 (2)	0.2019 (5)	0.09367 (19)	0.0580 (13)	
H21A	0.5617	0.2551	0.1275	0.087*	
H21B	0.5950	0.1393	0.0897	0.087*	
H21C	0.5116	0.1420	0.0935	0.087*	

### Atomic displacement parameters (Å^2^)

**Table:**

	*U*^11^	*U*^22^	*U*^33^	*U*^12^	*U*^13^	*U*^23^
Ni1	0.0294 (3)	0.0185 (3)	0.0161 (3)	−0.0020 (3)	0.0053 (2)	−0.0006 (2)
S1	0.0359 (4)	0.0216 (5)	0.0173 (4)	−0.0002 (4)	0.0042 (3)	−0.0015 (3)
S2	0.0332 (5)	0.0450 (7)	0.0512 (6)	0.0053 (5)	0.0129 (4)	0.0132 (5)
N1	0.053 (2)	0.048 (2)	0.0219 (15)	0.0008 (17)	0.0073 (14)	−0.0063 (14)
N2	0.0343 (15)	0.0295 (18)	0.0192 (13)	−0.0021 (13)	0.0053 (11)	−0.0032 (12)
N3	0.0378 (16)	0.0256 (17)	0.0202 (13)	0.0056 (13)	0.0041 (11)	−0.0039 (12)
N4	0.0314 (14)	0.0211 (16)	0.0166 (12)	−0.0005 (12)	0.0059 (11)	−0.0036 (11)
N5	0.0259 (14)	0.0213 (16)	0.0192 (12)	−0.0018 (12)	0.0069 (10)	−0.0026 (11)
N6	0.0275 (14)	0.0239 (17)	0.0255 (14)	0.0023 (12)	0.0028 (11)	−0.0027 (12)
O1	0.0355 (13)	0.0473 (18)	0.0255 (12)	−0.0032 (12)	0.0031 (10)	0.0014 (11)
C1	0.049 (2)	0.039 (2)	0.0228 (17)	0.0024 (18)	0.0068 (16)	−0.0055 (16)
C2	0.039 (2)	0.039 (3)	0.0268 (18)	−0.0054 (18)	0.0087 (15)	0.0032 (17)
C3	0.043 (2)	0.049 (3)	0.0314 (19)	−0.002 (2)	0.0063 (17)	0.0078 (18)
C4	0.040 (2)	0.043 (3)	0.047 (2)	−0.0003 (19)	−0.0002 (18)	0.012 (2)
C5	0.040 (2)	0.042 (3)	0.054 (2)	0.0023 (19)	0.0148 (19)	0.004 (2)
C6	0.0386 (19)	0.036 (2)	0.0304 (18)	−0.0014 (17)	0.0104 (15)	0.0004 (16)
C7	0.0352 (19)	0.037 (2)	0.0235 (16)	−0.0067 (17)	0.0086 (14)	0.0000 (15)
C8	0.040 (2)	0.035 (2)	0.0218 (16)	0.0003 (17)	0.0072 (15)	−0.0051 (15)
C9	0.0378 (19)	0.030 (2)	0.0249 (17)	0.0000 (16)	0.0079 (14)	−0.0034 (15)
C10	0.0269 (16)	0.024 (2)	0.0214 (15)	−0.0052 (14)	0.0077 (13)	−0.0041 (14)
C11	0.0260 (16)	0.025 (2)	0.0210 (15)	−0.0025 (14)	0.0059 (13)	−0.0033 (14)
C12	0.0243 (16)	0.025 (2)	0.0221 (15)	−0.0018 (14)	0.0040 (12)	−0.0026 (14)
C13	0.0241 (15)	0.022 (2)	0.0241 (16)	0.0008 (14)	0.0030 (12)	0.0015 (14)
C14	0.0211 (16)	0.033 (2)	0.0285 (17)	−0.0031 (14)	0.0039 (13)	0.0019 (15)
C15	0.0316 (19)	0.034 (2)	0.0370 (19)	−0.0002 (16)	−0.0006 (15)	0.0077 (17)
C16	0.035 (2)	0.050 (3)	0.036 (2)	−0.0012 (19)	−0.0063 (16)	0.0151 (19)
C17	0.043 (2)	0.050 (3)	0.0260 (18)	−0.006 (2)	−0.0013 (15)	0.0019 (18)
C18	0.0357 (19)	0.039 (2)	0.0247 (17)	−0.0036 (17)	0.0024 (15)	−0.0016 (16)
C19	0.0243 (16)	0.024 (2)	0.0280 (17)	−0.0036 (13)	0.0051 (13)	0.0024 (14)
C20	0.042 (2)	0.064 (3)	0.077 (3)	−0.018 (2)	−0.005 (2)	−0.012 (3)
C21	0.049 (2)	0.053 (3)	0.076 (3)	0.010 (2)	0.026 (2)	0.031 (2)

### Geometric parameters (Å, °)

**Table:**

Ni1—N5^i^	1.906 (3)	C5—C6	1.386 (5)
Ni1—N5	1.906 (3)	C5—H5	0.9500
Ni1—S1^i^	2.1748 (8)	C6—C7	1.396 (5)
Ni1—S1	2.1748 (8)	C6—H6	0.9500
S1—C10	1.726 (3)	C7—C8	1.434 (5)
S2—O1	1.496 (2)	C8—C9	1.438 (5)
S2—C20	1.790 (5)	C9—H9	0.9500
S2—C21	1.794 (4)	C11—C12	1.420 (5)
N1—C2	1.356 (5)	C11—H11	0.9500
N1—C1	1.362 (4)	C12—C13	1.386 (4)
N1—H1N	0.8800	C12—C19	1.456 (4)
N2—C9	1.293 (4)	C13—H13	0.9500
N2—N3	1.374 (4)	C14—C15	1.394 (5)
N3—C10	1.369 (4)	C14—C19	1.400 (5)
N3—H3N	0.8800	C15—C16	1.377 (5)
N4—C10	1.298 (4)	C15—H15	0.9500
N4—N5	1.400 (3)	C16—C17	1.392 (6)
N5—C11	1.304 (4)	C16—H16	0.9500
N6—C13	1.345 (4)	C17—C18	1.372 (5)
N6—C14	1.384 (4)	C17—H17	0.9500
N6—H6N	0.8800	C18—C19	1.408 (4)
C1—C8	1.370 (5)	C18—H18	0.9500
C1—H1	0.9500	C20—H20A	0.9800
C2—C3	1.385 (5)	C20—H20B	0.9800
C2—C7	1.428 (4)	C20—H20C	0.9800
C3—C4	1.367 (6)	C21—H21A	0.9800
C3—H3	0.9500	C21—H21B	0.9800
C4—C5	1.401 (5)	C21—H21C	0.9800
C4—H4	0.9500		
			
N5^i^—Ni1—N5	180.000 (1)	C7—C8—C9	129.1 (3)
N5^i^—Ni1—S1^i^	86.25 (7)	N2—C9—C8	121.3 (3)
N5—Ni1—S1^i^	93.75 (7)	N2—C9—H9	119.3
N5^i^—Ni1—S1	93.75 (7)	C8—C9—H9	119.3
N5—Ni1—S1	86.25 (7)	N4—C10—N3	115.5 (3)
S1^i^—Ni1—S1	180.000 (1)	N4—C10—S1	125.0 (2)
C10—S1—Ni1	94.55 (10)	N3—C10—S1	119.5 (2)
O1—S2—C20	105.20 (18)	N5—C11—C12	129.6 (3)
O1—S2—C21	105.92 (18)	N5—C11—H11	115.2
C20—S2—C21	97.8 (3)	C12—C11—H11	115.2
C2—N1—C1	110.0 (3)	C13—C12—C11	131.2 (3)
C2—N1—H1N	125.0	C13—C12—C19	105.5 (3)
C1—N1—H1N	125.0	C11—C12—C19	123.3 (3)
C9—N2—N3	113.5 (3)	N6—C13—C12	110.1 (3)
C10—N3—N2	120.1 (3)	N6—C13—H13	124.9
C10—N3—H3N	119.9	C12—C13—H13	124.9
N2—N3—H3N	119.9	N6—C14—C15	130.1 (3)
C10—N4—N5	111.3 (3)	N6—C14—C19	107.2 (3)
C11—N5—N4	113.6 (3)	C15—C14—C19	122.7 (3)
C11—N5—Ni1	126.4 (2)	C16—C15—C14	116.7 (4)
N4—N5—Ni1	120.03 (19)	C16—C15—H15	121.6
C13—N6—C14	110.1 (3)	C14—C15—H15	121.6
C13—N6—H6N	125.0	C15—C16—C17	122.0 (3)
C14—N6—H6N	125.0	C15—C16—H16	119.0
N1—C1—C8	109.2 (4)	C17—C16—H16	119.0
N1—C1—H1	125.4	C18—C17—C16	121.2 (3)
C8—C1—H1	125.4	C18—C17—H17	119.4
N1—C2—C3	131.2 (3)	C16—C17—H17	119.4
N1—C2—C7	107.9 (3)	C17—C18—C19	118.6 (4)
C3—C2—C7	120.9 (4)	C17—C18—H18	120.7
C4—C3—C2	118.7 (4)	C19—C18—H18	120.7
C4—C3—H3	120.7	C14—C19—C18	118.8 (3)
C2—C3—H3	120.7	C14—C19—C12	107.1 (3)
C3—C4—C5	121.5 (4)	C18—C19—C12	134.1 (3)
C3—C4—H4	119.3	S2—C20—H20A	109.5
C5—C4—H4	119.3	S2—C20—H20B	109.5
C6—C5—C4	120.8 (4)	H20A—C20—H20B	109.5
C6—C5—H5	119.6	S2—C20—H20C	109.5
C4—C5—H5	119.6	H20A—C20—H20C	109.5
C5—C6—C7	118.8 (3)	H20B—C20—H20C	109.5
C5—C6—H6	120.6	S2—C21—H21A	109.5
C7—C6—H6	120.6	S2—C21—H21B	109.5
C6—C7—C2	119.3 (3)	H21A—C21—H21B	109.5
C6—C7—C8	135.2 (3)	S2—C21—H21C	109.5
C2—C7—C8	105.4 (3)	H21A—C21—H21C	109.5
C1—C8—C7	107.5 (3)	H21B—C21—H21C	109.5
C1—C8—C9	123.4 (4)		
			
N5^i^—Ni1—S1—C10	167.97 (12)	C7—C8—C9—N2	−8.1 (6)
N5—Ni1—S1—C10	−12.03 (12)	N5—N4—C10—N3	−178.4 (2)
C9—N2—N3—C10	−170.8 (3)	N5—N4—C10—S1	1.3 (4)
C10—N4—N5—C11	164.8 (3)	N2—N3—C10—N4	179.0 (3)
C10—N4—N5—Ni1	−14.8 (3)	N2—N3—C10—S1	−0.7 (4)
S1^i^—Ni1—N5—C11	17.7 (3)	Ni1—S1—C10—N4	9.5 (3)
S1—Ni1—N5—C11	−162.3 (3)	Ni1—S1—C10—N3	−170.8 (2)
S1^i^—Ni1—N5—N4	−162.8 (2)	N4—N5—C11—C12	−2.2 (5)
S1—Ni1—N5—N4	17.2 (2)	Ni1—N5—C11—C12	177.4 (2)
C2—N1—C1—C8	−1.6 (4)	N5—C11—C12—C13	−12.0 (6)
C1—N1—C2—C3	−179.1 (4)	N5—C11—C12—C19	170.0 (3)
C1—N1—C2—C7	0.7 (4)	C14—N6—C13—C12	−0.7 (4)
N1—C2—C3—C4	178.5 (4)	C11—C12—C13—N6	−178.3 (3)
C7—C2—C3—C4	−1.3 (6)	C19—C12—C13—N6	0.0 (4)
C2—C3—C4—C5	0.3 (6)	C13—N6—C14—C15	−176.7 (3)
C3—C4—C5—C6	0.0 (6)	C13—N6—C14—C19	1.1 (3)
C4—C5—C6—C7	0.7 (6)	N6—C14—C15—C16	177.8 (3)
C5—C6—C7—C2	−1.8 (5)	C19—C14—C15—C16	0.4 (5)
C5—C6—C7—C8	−179.1 (4)	C14—C15—C16—C17	0.9 (5)
N1—C2—C7—C6	−177.7 (3)	C15—C16—C17—C18	−0.9 (6)
C3—C2—C7—C6	2.1 (5)	C16—C17—C18—C19	−0.4 (5)
N1—C2—C7—C8	0.3 (4)	N6—C14—C19—C18	−179.5 (3)
C3—C2—C7—C8	−179.8 (3)	C15—C14—C19—C18	−1.6 (5)
N1—C1—C8—C7	1.8 (4)	N6—C14—C19—C12	−1.1 (3)
N1—C1—C8—C9	−176.2 (3)	C15—C14—C19—C12	176.9 (3)
C6—C7—C8—C1	176.3 (4)	C17—C18—C19—C14	1.5 (5)
C2—C7—C8—C1	−1.3 (4)	C17—C18—C19—C12	−176.4 (3)
C6—C7—C8—C9	−5.9 (7)	C13—C12—C19—C14	0.7 (3)
C2—C7—C8—C9	176.5 (3)	C11—C12—C19—C14	179.1 (3)
N3—N2—C9—C8	−179.2 (3)	C13—C12—C19—C18	178.8 (3)
C1—C8—C9—N2	169.4 (3)	C11—C12—C19—C18	−2.7 (6)

Symmetry codes: (i) −*x*+3/2, −*y*+3/2, −*z*+1.

### Hydrogen-bond geometry (Å, °)

**Table:**

*D*—H···*A*	*D*—H	H···*A*	*D*···*A*	*D*—H···*A*
N1—H1n···O1	0.88	2.10	2.890 (4)	148
N6—H6n···O1^ii^	0.88	2.03	2.855 (4)	156

Symmetry codes: (ii) −*x*+3/2, *y*−1/2, −*z*+1/2.
